# Biological characteristics of prostate cancer cells are regulated by hypoxia-inducible factor 1α

**DOI:** 10.3892/ol.2014.2259

**Published:** 2014-06-17

**Authors:** XIANG HUANG, JIANHUA ZHOU, JUNYAN LIU, BINZHI TANG, FENGYAN ZHAO, YI QU

**Affiliations:** 1 Department of Urology, Sichuan Academy of Medical Sciences and Sichuan Provincial People’s Hospital, Chengdu, Sichuan 610041, P.R. China; 2 Department of Pediatrics, West China Second University Hospital, Chengdu, Sichuan 610041, P.R. China; 3 Key Laboratory of Obstetric, Gynecologic and Pediatric Diseases and Birth Defects of Ministry of Education, Sichuan University, Chengdu, Sichuan 610041, P.R. China

**Keywords:** hypoxia inducible factor 1α, prostate cancer, proliferation, apoptosis, migration

## Abstract

Hypoxia-inducible factor (HIF)-1α has been reported to be associated with malignancy in a number of types of cancer. However, the role of HIF-1 α in the regulation of prostate cancer (PCa) growth has yet to be elucidated. The present study aimed to investigate the effect of HIF-1α on the biological characteristics of the PCa PC3 cell line. Full-length (fL) HIF-1α and dominant-negative (dn) HIF-1α were transfected into PC3 cells. The expression of HIF-1α and its downstream genes, including vascular endothelial growth factor (VEGF), erythropoietin (EPO) and CXC chemokine receptor 4 (CXCR4), were detected using western blot analysis. Cell proliferation, apoptosis and migration were assessed using MTT, terminal deoxynucleotidyl transferase-mediated dUTP nick end labeling and Boyden chamber assays. The expression of VEGF, EPO and CXCR4 was found to be upregulated in the fL HIF-1α-transfected PC3 cells and downregulated in the dn HIF-1α-transfected PC3 cells. The overexpression of HIF-1α was observed to enhance cell proliferation and migration and decrease docetaxol-induced cell apoptosis. However, dn HIF-1α was found to attenuate cell proliferation and migration, and promote docetaxol-induced cell apoptosis. These findings indicate that HIF-1α regulates the proliferation, apoptosis and migration of PC3 cells, at least in part, by regulating the expression of its target genes, including VEGF, EPO and CXCR4. Thus, the use of HIF-1α inhibitors may be a promising therapy for the treatment of PCa.

## Introduction

Hypoxia-inducible factor (HIF)-1 has been reported as a transcription factor important for regulating cell energy, angiogenesis, apoptosis, adhesion and growth (1–5). HIF-1 is a heterodimeric protein complex that is composed of α and β subunits. The α subunit of HIF-1 is the determinant of HIF-1 transcriptional activity and acts as a regulatory subunit. The regulation of HIF-1α is induced by a variety of factors, including oxygen tension, cytokines and oncogenes (6–8). Upon activation, HIF-1 interacts with the hypoxia response element of HIF-1 target genes and promotes their transcription (9). HIF-1α has been found to be overexpressed in several types of human cancer (10) and to enhance the adaptive response of cancer to oxygen deprivation through upregulating the expression of its target genes (11). Based on the association between HIF-1α and malignant cancer phenotypes, the inhibition of HIF-1α is becoming one of the most important strategies for cancer treatment (12).

Prostate cancer (PCa) is one of the most common types of malignant tumor worldwide. It has been reported that HIF-1α may be a key factor for determining cancer malignancy and prognosis (13). However, the role of HIF-1α in PCa has yet to be elucidated. The present study aimed to investigate the effect of HIF-1α on the biological characteristics of PCa cells, including cell proliferation, apoptosis and migration, through transfecting full length (fL) and dominant-negative (dn) HIF-1α into the PCa PC3 cell line.

## Materials and methods

### Plasmids

pcDNA3.1 plasmids were purchased from Invitrogen Life Technologies (Carlsbad, CA, USA). pcDNA3.1-fL HIF-1α (fL HIF-1α) and pcDNA3.1-dn HIF-1 α (dn HIF-1α) plasmids were then constructed.

### Cell culture and transfection

The PCa PC3 cell line was purchased from the American Type Culture Collection (Manassas, VA, USA) and cultured in RPMI-1640 supplemented with 10% fetal bovine serum and 1% L-glutamine. The cells were incubated at 37°C in 5% CO_2_ and 95% air. The cells were seeded onto six-well plates (Becton Dickinson, Franklin Lakes, NJ, USA) and maintained overnight to obtain ~80% confluence. Lipofectamine^®^ 2000 (Invitrogen, Rockville, MD, USA) was used for cell transfection according to the manufacturer’s instructions. Following selection with 400 μg/ml G418 (Invitrogen) for two weeks, separate positive clones of each transfection were grown and termed PC3-pcDNA3.1-fL HIF-1α (fL HIF-1α), PC3-pcDNA3.1-dn HIF-1α (dn HIF-1α) and PC3-pcDNA3.1 (pcDNA3.1). PC3-pcDNA3.1 and wild-type PC3 cells were used as controls in subsequent experiments.

### Western blot analysis

Cell lysates were obtained using radioimmunoprecipitation assay buffer (Sigma-Aldrich, St. Louis, MO, USA). A total of 10 μg protein was separated on 8% sodium dodecyl sulfate-polyacrylamide gels (Beijing Solarbio Science and Technology Co., Ltd., Beijing, China), transferred onto polyvinylidene difluoride membranes (Roche, Basel, Switzerland) and blocked using 5% blocking buffer (Wuhan Boster Biological Technology, Ltd., Wuhan, China). The membranes were washed and incubated with the following primary antibodies: Rabbit anti-human HIF-1α polyclonal (dilution, 1:150; Santa Cruz Biotechnology, Inc., Santa Cruz, CA, USA), mouse anti-human VEGF monoclonal (dilution, 1:200; Santa Cruz Biotechnology, Inc.), rabbit anti-human EPO polyclonal (dilution, 1:200; Santa Cruz Biotechnology, Inc.), rabbit anti-human CXCR4 polyclonal (dilution, 1:150; Abnova, Taipei, Taiwan) and rabbit anti-human β-actin polyclonal (dilution, 1:200; Santa Cruz Biotechnology, Inc.) for 1 h at room temperature and overnight at 4°C. Subsequent to being washed with Tris-buffered saline containing Tween 20, the membranes were incubated with horseradish peroxidase-conjugated secondary antibodies (dilution, 1:3,000; Zsgb-Bio, Beijing, China). The signals from the bound antibodies were identified using an enhanced chemiluminescence kit (Amersham, Freiburg, Germany). The integrated density value (IDV) of each band was assessed using a Gel-Pro Image Analyzer (Bio-Rad Laboratories, Inc., Hercules, CA, USA), and the ratios of the integrated density values (HIF-1α/β-actin, VEGF/β-actin, EPO/β-actin and CXCR4/β-actin) were calculated to assess the relative expression levels of the proteins.

### Cell proliferation detected using MTT assay

The PC3 cells were plated on 96-well plates and maintained in normoxic conditions for five days. The viability of the PC3 cells was assessed using an MTT assay. In brief, 20 μl MTT solution (5 g/l; Sigma-Aldrich) was added to each well and incubated for 4 h at 37°C. A total of 150 ml dimethyl sulfoxide (Sigma-Aldrich) was then added to dissolve the crystal once the growth medium was removed. The absorbance of 200 μl of solution from each well was measured at 492 nm using a microplate reader (Thermo Fisher Scientific, Waltham, MA, USA). Cell growth curves were generated based on the corresponding optical density values at 492 nm.

### Apoptosis assay

Exponential phase cells were split at a density of 5×10^4^ cells per well on 96-well plates (Becton Dickinson). Subsequent to 12 h of incubation, the RPMI-1640 medium was replaced with RPMI-1640 containing 2 or 4 μmol/l docetaxol (Aventis Pharma, Ltd., Mumbai, India) for 48 h. Cell apoptosis was detected using an *in situ* terminal deoxynucleotidyl transferase-mediated dUTP nick end labeling (TUNEL) kit (Roche) according to the manufacturer’s instructions. A total of 10 fields were chosen randomly at ×400 magnification to count the numbers of apoptotic and total cells. The apoptotic index (AI) was calculated as follows: AI = (number of apoptotic cells / total number counted) × 100.

### In vitro migration assay

The cells were harvested through trypsinization, then counted and resuspended in RPMI-1640 at a concentration of 1×10^5^/ml. Next, 0.5-ml aliquots of cell suspension were added to the upper chamber of Millicell^®^ Inserts (Millipore Corporation, Billerica, MA, USA). The upper and lower chambers were separated using a 12-mm pore polycarbonate membrane that was coated with Matrigel™ (Becton Dickinson). Subsequent to 24 h of incubation at 37°C, the remaining cells on the upper side of the chamber were removed using a cotton swab. The cells that had migrated through the pores to the bottom side of the membrane were fixed using 3.7% paraformaldehyde and stained with hematoxylin and eosin. The number of migrated cells was counted in 10 randomly selected fields using a microscope.

### Statistical analysis

Student’s t-test was used to compare two groups. Analysis of variance with Fisher’s post-hoc test was used for comparing more than two groups. P<0.05 was considered to indicate a statistically significant difference.

## Results

### dn HIF-1α inactivates HIF-1 and attenuates the expression of HIF-1 downstream genes

**Figure 1:**
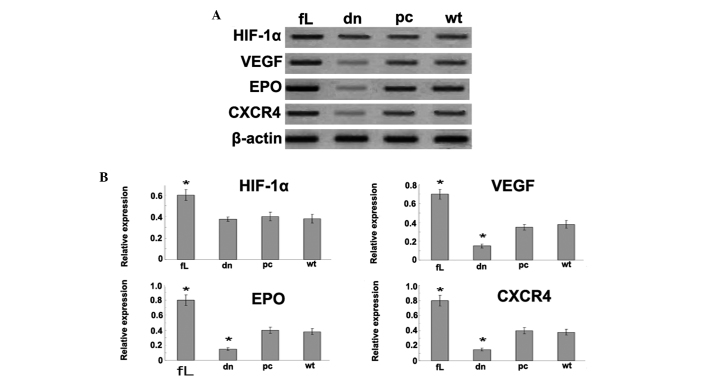
Expression of HIF-1α and its downstream genes, VEGF, EPO and CXCR4. (A) Western blot analysis was used to detect the effect of dn HIF-1α and fL HIF-1α on VEGF, EPO and CXCR4 expression. β-actin was used as a loading control. VEGF, EPO and CXCR4 were upregulated by fL HIF-1α, while dn HIF-1α significantly downregulated the HIF-1 target genes. (B) The relative protein expression of HIF-1α, VEGF, EPO and CXCR4 was calculated as integrated density values. Data are presented as the mean ± standard error of the mean from three independent experiments. ^*^P<0.05 vs. pcDNA3.1 or wild type. HIF, hypoxia-inducible factor; VEGF, vascular endothelial growth factor; EPO, erythropoietin; CXCR4, CXC chemokine receptor 4; fL, full length; dn, dominant-negative; pc, pcDNA3.1; wt, wild-type.

The effect of dn HIF-1α and fL HIF-1α on VEGF, EPO and CXCR4 expression was analyzed using western blot analysis. VEGF, EPO and CXCR4 were observed to be upregulated by fL HIF-1α, while dn HIF-1α was found to downregulate the HIF-1 target genes, with no impact on HIF-1α expression (Fig. 1).

### dn HIF-1α inhibits PC3 proliferation

**Figure 2:**
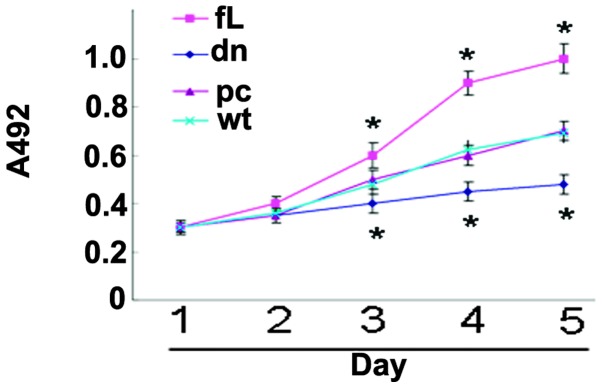
HIF-1α regulates PC3 cell growth. Viable cell numbers were estimated using a colorimetric MTT assay at the different incubation time-points. The proliferation of the fL HIF-1α transfectants was significantly enhanced, while the proliferation of the dn HIF-1α transfectants was significantly suppressed compared with the control cells. ^*^P<0.05 vs. control cells. HIF, hypoxia-inducible factor; fL, full length; dn, dominant-negative; pc, pcDNA3.1; wt, wild-type.

Compared with the control cells, the proliferation of the fL HIF-1α transfectants was observed to be significantly enhanced, while the proliferation of the dn HIF-1α transfectants was found to be significantly suppressed (Fig. 2).

### HIF-1α attenuates docetaxol-induced PC3 cell apoptosis

**Figure 3:**
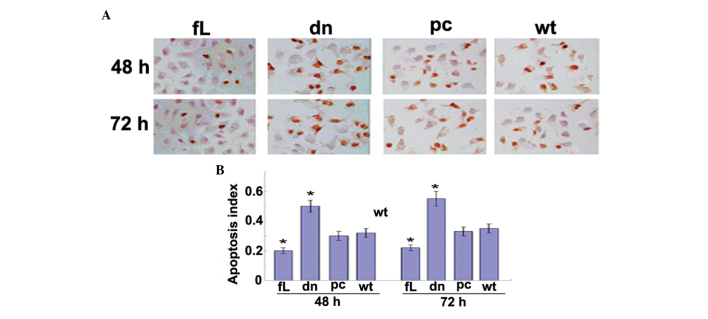
HIF-1α activation prevents docetaxol-induced tumor apoptosis. (A) The terminal deoxynucleotidyl transferase-mediated dUTP nick end labeling (TUNEL) assay was used to detect PC3 cell apoptosis, and cells with yellow or brown granules confined to the nucleus were defined as apoptotic cells (magnification, ×400). (B) The apoptotic index was calculated and showed that HIF-1α protected PC3 cells from docetaxol-induced apoptosis, while dn HIF-1α was associated with increased apoptosis. ^*^P<0.05 vs. controls. HIF, hypoxia-inducible factor; fL, full length; dn, dominant-negative; pc, pcDNA3.1; wt, wild-type.

A TUNEL assay was used to detect apoptosis in the PC3 cells subjected to docetaxol treatment. Following treatment with docetaxol for 48 and 72 h, dn HIF-1α was found to promote PC3 cell apoptosis, while fL HIF-1α was observed to have an anti-apoptotic effect (Fig. 3).

### HIF-1α enhances PC3 cell migration

**Figure 4:**
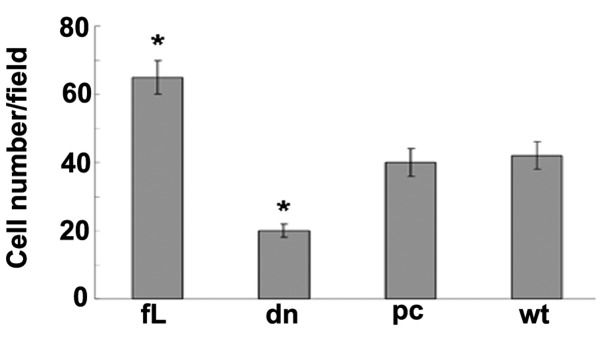
Effect of HIF-1α on the promotion of PC3 cell migration. A Boyden chamber assay was used to detect cell migration. The migration of the PC3 cells from the upper chamber to the lower chamber was analyzed 24 h after cell seeding. The cells that had migrated through the pores to the bottom side of the membrane were fixed and stained with hematoxylin and eosin. The number of migrating cells was counted in 10 randomly selected fields using light microscopy. Data are presented as the mean ± standard deviation of two independent experiments performed in triplicate. dn HIF-1α significantly inhibited PC3 migration, while the number of cells that migrated to the lower chamber in the fL HIF-1α group was increased compared with that in the control groups. ^*^P<0.05 vs. control groups. HIF, hypoxia-inducible factor; fL, full length; dn, dominant-negative; pc, pcDNA3.1; wt, wild-type.

A Boyden chamber assay was used to detect cell migration, and revealed that the PC3 cells transfected with fL HIF-1α migrated more rapidly compared with those transfected with dn HIF-1α and the control cells (Fig. 4).

## Discussion

dn HIF-1α is a truncated variant of HIF-1α that functions in a dn manner. Through competing with normal fL HIF-1α for the binding site of HIF-1β, dn HIF-1α inhibits the formation of functional HIF-1 (14). In the present study, a significant increase in HIF-1α expression was observed in the pcDNA3.1-fL HIF-1α PC3 cells, but not in the pcDNA3.1-dn HIF-1α PC3 cells. Furthermore, the expression of the HIF-1α target genes, VEGF, EPO and CXCR4, was found to be increased in the pcDNA3.1-fL HIF-1α-transfected PC3 cells, but decreased in the pcDNA3.1-dn HIF-1α-transfected PC3 cells.

VEGF has a vital role in the growth of blood vessels. Although the majority of studies have been focused on the function of VEGF and VEGF receptors in angiogenesis and in endothelial cells, the role of VEGF in carcinogenesis may be another function that requires highlighting. VEGF has been reported to have a role in the regulation of a number of functions in tumor cells, including survival, adhesion, migration and invasion (15). Similarly, EPO has also been reported to have other effects in addition to its role in erythropoiesis, including the promotion of tumor cell growth and survival (16). The CXCR4 signaling pathway is crucial in modulating cancer cell migration. The activation of CXCR4 leads to cellular skeleton remolding and pseudopodia formation, and consequently enhanced cell mobility and migration (17). Therefore, the HIF-1α-induced promotion of cell growth and migration and the reversal of docetaxol-induced cell apoptosis, may be associated with the overexpression of the HIF-1α downstream effectors VEGF, EPO and CXCR4.

Docetaxol is one of the most common drugs for the chemotherapeutic treatment of PCa (18). In the present study, following treatment with docetaxol, dn HIF-1α was found to promote PC3 apoptosis, while fL HIF-1α exhibited an anti-apoptotic effect. These findings indicate that dn HIF-1α may make PC3 cells more susceptible to docetaxol-induced apoptosis and that fL HIF-1α may rescue this susceptibility.

HIF-1α has a central role in regulating tumor behavior, including proliferation, apoptosis and migration, thus the inhibition of the HIF-1α pathway may be a promising strategy for attenuating tumor progression. Several methods to inhibit HIF-1α have been investigated, including using HIF-1α small interfering RNA to inhibit HIF-1α transcription, agents to accelerate the proteasome-dependent degradation of the HIF-1α protein and small molecular inhibitors to block the transcriptional activity of HIF-1α (19,20). The dn HIF-1α-induced inhibition of HIF-1α has been reported in a number of types of cancer, including malignant gliomas, gastric cancer and breast carcinoma, however, the underlying mechanism has yet to be elucidated (21–23). The present study showed that HIF-1α regulates certain biological characteristics of PC3 cells and that dn HIF-1α may be a promising biotherapy for PCa, providing novel insight into our understanding of the mechanism underlying the carcinogenesis of PCa and a potential therapy to treat PCa.
